# To share or hide under performance pressure: the role of supervisor support in shaping subordinate knowledge management behaviors

**DOI:** 10.3389/fpsyg.2025.1586812

**Published:** 2025-05-27

**Authors:** Jia Xu, Jingrong Chen, Qisi Lan, Man Zhao

**Affiliations:** ^1^School of Marxism, University of Electronic Science and Technology of China, Chengdu, Sichuan, China; ^2^Department of Education Evaluation and Supervision, Chengdu University of Technology, Chengdu, Sichuan, China; ^3^Jinshan College of Fujian Agriculture and Forestry University, Fuzhou, China; ^4^School of Internet Economics and Business, Fujian University of Technology, Fuzhou, Fujian, China; ^5^School of Management, Yunnan Normal University, Kunming, Yunnan, China

**Keywords:** employee performance pressure, perceived supervisor support, knowledge sharing, knowledge hiding, conservation of resource theory

## Abstract

**Introduction:**

While knowledge sharing is widely acknowledged as a critical driver of organizational learning and development, employees may engage in knowledge hiding—particularly under performance-related pressure. This behavior can impede organizational growth, underscoring the need to understand the boundary conditions that determine whether performance pressure leads to knowledge sharing or knowledge hiding. Grounded in conservation of resources theory, this study proposes a dual-path model that examines how perceived supervisor support moderates the effects of employee performance pressure on knowledge management behaviors.

**Methods:**

Two studies were conducted to test the proposed model. Study 1 employed a scenario-based experimental design, while Study 2 used a multi-wave survey approach. Both methods assessed the interaction between performance pressure and perceived supervisor support in predicting knowledge sharing and hiding behaviors.

**Results:**

Findings revealed two distinct paths. The enrichment path was supported: when employees perceived high levels of supervisor support, performance pressure positively influenced their knowledge sharing behavior. Conversely, the depletion path was also confirmed: when supervisor support was perceived as low, performance pressure significantly increased tendencies toward knowledge hiding.

**Discussion:**

This study advances understanding of the nuanced relationship between performance pressure and knowledge management behaviors by identifying perceived supervisor support as a key boundary condition. The dual-path model offers theoretical insights into how resource dynamics influence employee responses to stress, with practical implications for fostering positive knowledge behaviors in high-pressure work environments.

## Introduction

Knowledge management behaviors, such as knowledge sharing and hiding, are crucial for organizational learning and growth (Casimir et al., 2012; Connelly et al., 2012; Montani et al., 2024). Knowledge sharing, defined as the behavior through which employees disseminate their expertise and experiences, enhances the overall knowledge base and capabilities of an organization (Lu et al., 2006). When employees share their knowledge, others can learn and apply this information, leading to the increase on productivity and problem-solving abilities (Hansen et al., 2005; Kim et al., 2021; Yao et al., 2023). Moreover, research confirmed that knowledge sharing fostered innovative behaviors through facilitating the exchange of diverse perspectives and experiences (Hansen et al., 2005; Kim et al., 2021; Yao et al., 2023). Despite the benefits of knowledge sharing, employees often engage in knowledge hiding behaviors, which can significantly impede organizational development. Knowledge hiding refers to the intentional concealment of knowledge from others, thereby limiting its dissemination and utilization (Connelly et al., 2019; Connelly et al., 2012). This behavior creates information asymmetry among employees, hindering teamwork and innovation and ultimately reducing organizational performance (Khoreva and Wechtler, 2020; Nguyen et al., 2022). Consequently, organizations continuously seek ways to encourage positive knowledge management behaviors to enhance individual, team, and organizational effectiveness (Hansen et al., 2005; Kim et al., 2021; Yao et al., 2023). Considering the importance of employee knowledge management behaviors, previous research exerts endeavors into why and when knowledge sharing and hiding occurs and has established that both individual and workplace factors are closely related to knowledge management behaviors (He et al., 2024; Li et al., 2023; Mahapatra and Ford, 2024; Shujahat et al., 2024; Skerlavaj et al., 2018; Sofyan et al., 2023; Zhang et al., 2022). Among these antecedents, pressure-related elements have emerged as the critical factor influencing employee knowledge management behaviors (He et al., 2024; Montani et al., 2024; Skerlavaj et al., 2018; Sofyan et al., 2023; Zhang et al., 2022). For instance, challenge-nature pressure shows positive impacts on knowledge sharing and also inhibits hiding, while hindrance-nature pressure leads to more knowledge hiding and less sharing (Montani et al., 2024; Skerlavaj et al., 2018).

However, prior research barely captures the performance pressure as the key factor in liking knowledge management behaviors. In light of the intensification of information technology and workplace competition, employees are confronted with heightened performance pressure (Mitchell et al., 2019; Spoelma, 2022; Wang et al., 2025), it becomes important for managers to consider how employee management knowledge behaviors under high performance pressure context. In addition, organizations often use performance pressure to motivate employees to improve their performance, which inevitably involves managing knowledge management behaviors under such conditions. Performance pressure is the subjective experience of employees feeling the need to enhance their work performance to avoid significant consequences (Mitchell et al., 2018; Mitchell et al., 2019). Unlike general stressors, performance pressure is uniquely tied to potential rewards such as promotions and salary increases, or negative outcomes like demotions and job loss (Mitchell et al., 2018; Mitchell et al., 2019). Therefore, research on performance pressure has shown the paradoxical effects on employee outcomes. For example, performance pressure can serve as a motivator, driving employees to perform better and engage in OCBs while it can also lead to burnout and counterproductive behaviors (Mitchell et al., 2018; Mitchell et al., 2019; Wang et al., 2025; Zhu et al., 2023). These findings suggest that the relationship between performance pressure and knowledge management behaviors is complex and multifaceted, indicating that there may be certain boundary conditions contributing to the emergence of knowledge sharing and knowledge hiding behaviors and that the interaction between driving factors and contextual factors must be considered.

To address these complexities, this study draws on Conservation of Resources (COR) theory (Hobfoll, 2002; Hobfoll et al., 2018; Lin et al., 2020) to develop a dual-path model to capture the enrichment and depletion effects of employee performance pressure interacting with their perception of supervisor support on knowledge sharing and hiding behaviors. Specifically, COR theory posits that individuals strive to acquire, retain, and protect their resources and the availability of resources can either enrich or deplete an individual’s capacity to cope with stressors (Hobfoll, 1989, 2002; Hobfoll et al., 2018). We thus introduce employees’ perception of supervisor support, a critical resource in the workplace, that can play a pivotal role in the effects of performance pressure on knowledge management behaviors. Specifically, in the enrichment path, we propose that in the high levels of perceived supervisor support, employees believe they own more necessary resources (Eisenberger et al., 2002; Holland et al., 2017; Nahum-Shani et al., 2014) to handle performance pressure effectively, thereby facilitating knowledge sharing. Conversely, the depletion path suggests low levels of perceived supervisor support can exacerbate the negative effects of performance pressure, leading to knowledge hiding.

This study contributes to the knowledge management literature by deepening our understanding of the intricate relationships between performance pressure, supervisor support, and knowledge management behaviors. First, we combine the dual perspectives of resource depletion and resource abundance to reveal the contextual dependency of performance pressure on knowledge sharing and knowledge hiding and then contribute to a deeper understanding of how contextual factors shape the impact of performance pressure on knowledge management behaviors. Second, this research expands the understanding of the drivers of knowledge sharing and knowledge hiding by examining the interaction between supervisor support and performance pressure, which can provide a more nuanced view of how managerial factors interact with performance pressure to drive knowledge management outcomes. Finally, this study further clarifies the paradoxical nature of performance pressure, highlighting its dual potential for both positive and negative outcomes and thus expanding the understanding of the situational factors that shape the paradoxical outcomes of performance pressure.

## Theory and hypotheses

According to COR theory (Hobfoll, 1989), individuals will strive to acquire, maintain, and protect the resources they value. These resources include material resources (such as money and time), conditional resources (such as stable jobs), personal traits (such as self-esteem), and energy resources (such as energy). In the workplace, performance pressure refers to the subjective experience that employees must increase their performance efforts or face significant consequences (Mitchell et al., 2018; Mitchell et al., 2019). Employees who meet performance demands will receive benefits, otherwise they will face negative consequences (Mitchell et al., 2018). Performance pressure thus has paradoxical impacts on psychological and behavioral outcomes (Kundi et al., 2022; Mitchell et al., 2019; Spoelma, 2022). For instance, performance pressure may drive employees to adopt conservative strategies to conserve resources (self-protection path) and then increase self-serving behaviors (Chen and Chen, 2023; Mitchell et al., 2018; Zhu et al., 2023), while it can also motivate employees to be more efficient and focused (self-motivation path), potentially enhancing OCB and engagement (Guo et al., 2024; Kundi et al., 2022; Mitchell et al., 2019).

Knowledge management behaviors mainly include sharing and hiding acts, which correspond to the dual-path consequences of performance pressure. The perspective of resource depletion (Hobfoll, 2002; Hobfoll et al., 2018; Lin et al., 2020) indicates performance pressure as a resource drain that will force employees to adopt conservative strategies, prioritizing the retention of resources to cope with work pressure (Julie et al., 2021; Kundi et al., 2022; Mitchell et al., 2018), thereby reducing knowledge sharing or increasing knowledge hiding. However, the enrichment-based perspective (Hobfoll, 2002; Hobfoll et al., 2018; Lin et al., 2020) states performance pressure can motivate employees to be more efficient and focused on their work (Guo et al., 2024; Mitchell et al., 2019), thereby increasing the potential for knowledge sharing or buffer knowledge hiding. Considering that knowledge sharing and hiding are independent, we suggest that the impact of performance pressure on knowledge management behaviors is context-dependent and that the impact of performance pressure on knowledge sharing and knowledge hiding varies depending on the specific conditions (Halbesleben et al., 2014; Hobfoll, 1989; Hobfoll et al., 2018).

### Performance pressure, supervisor support and knowledge sharing

In this study, we draw on the conservation of resources theory to identify the crucial role of perceived supervisor support in the relationship between performance pressure and knowledge management behaviors (Halbesleben et al., 2014; Hobfoll, 1989; Hobfoll et al., 2018). Perceived supervisor support refers to the extent to which employees perceive their supervisors as valuing their contributions and caring about their well-being (Eisenberger et al., 2002; Lee et al., 2023; Paustian-Underdahl et al., 2024). Because supervisors act as agents of the organization, they play a crucial role in helping employees obtain resources in the workplace (Eisenberger et al., 2002). For employees facing performance pressure, achieving performance goals and avoiding negative outcomes is their priority (Eisenberger and Aselage, 2009; Gardner, 2012; Mitchell et al., 2018; Mitchell et al., 2019). Performance pressure creates a resource-scarce environment where employees feel compelled to conserve their limited resources to meet high performance demands (Kundi et al., 2022; Zhu et al., 2023). Knowledge sharing, while beneficial for the organization, requires employees to invest time, effort, and expertise resources (Casimir et al., 2012; Huang et al., 2013) that may be perceived as scarce under performance pressure. However, the relationship between performance pressure and knowledge sharing remains uncertain and limited. Without external intervention, employees cannot determine whether knowledge sharing behaviors will bring them resource gains or losses, making it difficult for them to justify such actions. We thus suggest that supervisor support plays a crucial role in this relationship by providing the necessary reassurance and resources (Buch et al., 2015; Chae et al., 2019; Eisenberger et al., 2002). Supervisors who actively recognize and reward knowledge sharing efforts can mitigate employees’ concerns about resource depletion and encourage more proactive knowledge management behaviors (Buch et al., 2015; Montani et al., 2024; Wang et al., 2015). Moreover, supervisor support can create a positive work environment where employees feel valued and supported (Erdogan et al., 2024; Nahum-Shani et al., 2014; Shanock and Eisenberger, 2006), and thus decline the resource-related uncertainty associated with performance pressure. In such an environment, employees are more likely to see knowledge sharing not only as a resource investment but also to enhance their own and their colleagues’ performance, ultimately contributing to the overall success of the organization.

Specifically, when employees feel highly supported by their supervisors, they believe that their resources have been replenished and protected because high support can provide a resource-rich environment, making them feel supported and recognized (Beenen et al., 2017; Byrne et al., 2012; Holland et al., 2017; Sonnentag et al., 2024; Tews et al., 2020). From the perspective of resource gain (Halbesleben et al., 2014; Hobfoll, 1989; Hobfoll et al., 2018; Lin et al., 2020), under the support of supervisors, knowledge sharing behaviors are not only a form of resource investment but also a process of resource acquisition (Buch et al., 2015; Eisenberger et al., 2002), effectively addressing the demands brought about by performance pressure. First, in a resource gain environment (i.e., high supervisor support context), supervisors often recognize and reward employees who actively engage in knowledge sharing. For employees under performance pressure, this recognition can lead to positive performance evaluations, facilitating the achievement of performance goals. In addition, when employees share their knowledge and experiences, they can obtain new knowledge and insights from their colleagues. This exchange and complementarity of knowledge can enhance employees’ knowledge levels and professional skills, enabling them to better accomplish performance tasks and goals (Capatina et al., 2024; Henttonen et al., 2016; Kim and Yun, 2015; Lee et al., 2020). Thus, employees are more likely to engage in knowledge sharing under performance pressure with higher supervisor support. We hypothesized:

*Hypothesis 1: Performance pressure interacting with supervisor support can impact knowledge sharing, such that performance pressure can promote knowledge sharing when supervisors show higher support.*

### Performance pressure, supervisor support and knowledge hiding

Employees facing performance pressure are often in a state of self-protection because they worry/fear that failing to meet performance targets might threaten their position within the organization (Guo et al., 2024; Mitchell et al., 2018; Wang et al., 2025). Knowledge hiding is the deliberate behavior of withholding or concealing knowledge that one possesses, often driven by self-protection, distrust of colleagues, or fear of losing a competitive edge (Connelly et al., 2019; Connelly et al., 2012; Yao et al., 2023). From the perspective of resource depletion (Halbesleben et al., 2014; Hobfoll, 1989; Hobfoll et al., 2018; Lin et al., 2020), employees under performance pressure may view knowledge hiding as a strategy to conserve their limited resources. However, this approach does not necessarily help them achieve their performance goals and can leave them vulnerable to negative consequences due to unmet targets. Therefore, while knowledge hiding might initially seem like a coping mechanism for dealing with performance pressure, its effectiveness in achieving performance goals is questionable. This uncertainty highlights the need to consider other factors influencing this relationship. One such factor is the level of supervisor support. High levels of supervisor support can mitigate the effects of performance pressure by providing employees with the necessary resources, recognition, and guidance (Beenen et al., 2017; Byrne et al., 2012; Holland et al., 2017; Sonnentag et al., 2024; Tews et al., 2020). Conversely, low levels of supervisor support can exacerbate performance pressure, making knowledge hiding appear more attractive as a protective measure.

Specifically, from the perspective of resource depletion (Halbesleben et al., 2014; Hobfoll, 1989; Hobfoll et al., 2018; Lin et al., 2020), in the context of low supervisor support, employees may feel that their resources are not adequately replenished or protected (Eisenberger et al., 2002). This lack of support creates a resource-scarce environment where employees perceive that they do not have enough resources to meet their job demands (Erdogan et al., 2024; Sonnentag et al., 2024). Performance pressure, which expresses the urgency to achieve high performance (Eisenberger and Aselage, 2009; Mitchell et al., 2018), exacerbates this perception of resource scarcity by increasing the demand on employees without offering sufficient replenishment. Employees, therefore, prioritize conserving their remaining resources to cope with these demands. Knowledge hiding, in this context, becomes a strategy for employees to protect their limited resources (Bari et al., 2020; Connelly et al., 2012; Montani et al., 2024). When supervisors provide low levels of support, employees often feel that their efforts are neither recognized nor appreciated. This lack of recognition and appreciation can further diminish employees’ motivation to share knowledge, as they perceive it as an additional drain on their already limited resources.

Moreover, low supervisor support often translates into a lack of recognition and appreciation for employees’ efforts (Erdogan et al., 2024; Sonnentag et al., 2024). Employees facing performance pressure may feel compelled to preserve their competitive advantage by safeguarding their energy and time. In such an environment, knowledge hiding emerges to maintain personal resources and reduce the risk of overextension. Thus, performance pressure, combined with low supervisor support, creates a situation where knowledge hiding becomes an attractive option for employees to manage their limited resources and respond to performance pressure. We hypothesized:

*Hypothesis 2: Performance pressure interacting with supervisor support can impact knowledge sharing, such that performance pressure can promote knowledge hiding when supervisors show lower support.*

## Overview of studies

First, we tested our hypotheses by conducting a scenario-based experiment (Study 1) on part-time undergraduate students at a university in southwestern China. Next, we replicated our findings by conducting a time-lagged field survey of full-time employees from a manufacturing firm that emphasizes performance pressure on their employees (Study 2). The combination of these two studies not only confirmed the reliability of our measurements but also enabled us to verify both the internal and external validity of our research (Liu et al., 2021).

## Study 1: a scenario experiment

### Sample and procedures

A total of 361 part-time undergraduate students (53.19% female; 63.16% had obtained at least a college’s degree; *M*_age_ = 34.61 years, *SD* = 8.44; *M*_work tenure_ = 8.06, *SD* = 9.33) who came from a public university in China and had prior work experience in various companies were recruited to participate in our scenario experiment. Before the experiment commenced, we informed the participants that their participation was voluntary and guaranteed the confidentiality of their responses.

We employed a 2 (employee performance pressure: high vs. low) × 2 (perceived supervisor support: high vs. low) two-factor between-subjects design. Participants were first asked to report their demographic information and randomly assigned to one of the four experimental conditions, and then read the experimental scenarios. All participants were instructed to imagine themselves as Zhang Feng, a software development employee of a well-known high-tech company. Zhang Feng is working on an innovative machine-learning project with enormous market potential. In the high-level performance pressure condition, Zhang Feng faces significant pressure to meet the project goals. Conversely, in the low-level performance pressure condition, Zhang Feng is encouraged to prioritize personal development over the pursuit of high performance. In addition, in the high-level perception of supervisor support condition, Zhang Feng’s supervisor can provide care in his work and attach great importance to his own ideas. Conversely, in the low-level perception of supervisor support condition, Zhang Feng’s supervisor lacks concern for him in their work and does not value his well-being in the workplace.

After reading all scenarios (for full text, see Appendix), participants immediately completed the manipulation checks and then completed measures for knowledge sharing and knowledge hiding. These measures derive from role-play scenarios, allowing participants to respond according to the perceptions and senses triggered by the scenarios rather than their actual circumstances (Zhang et al., 2021). Finally, we asked participants to briefly describe the experiment’s purpose to assess their awareness of the manipulation (Yu and Duffy, 2021); however, none were able to provide an accurate response. This procedure confirmed the validity of our experiment.

### Measures

All measures were originally in English. To ensure meaning equivalence, we utilized the translation-back translation procedure to translate the English measures into simplified Mandarin Chinese (Brislin, 1986). Specifically, first, a scholar in the field of management was invited to translate the English items into simplified Mandarin Chinese. Second, two bilingual researchers in the same domain provided some feedback on the translated items to enhance the accuracy and clarity of the translation. Following this, minor modifications to the wording of the items were made based on their suggestions. Then another management scholar translated the items back into English. Ultimately, all research team members deliberated on any inconsistencies to achieve agreement on the final simplified Mandarin Chinese items. All items were rated using a seven-point Likert-type scale ranging from 1 (strongly disagree) to 7 (strongly agree). Table 1 reported the descriptive statistics, their correlation, and Cronbach’s alphas for the studied variables.

**Table 1:** Descriptive statistics and correlations among study variables in Study 1.

Variables	Mean	*SD*	1	2	3	4	5	6	7	8
1. Subordinate gender	0.47	0.50								
2. Subordinate age	34.61	8.44	0.10							
3. Subordinate tenure	8.06	9.33	0.08	0.48^**^						
4. Subordinate education	1.77	0.69	−0.03	−0.08	−0.01					
5. Performance pressure	4.49	1.23	0.08	0.06	0.05	0.01	(0.90)			
6. Supervisor support	3.95	1.37	0.04	0.13^*^	0.00	−0.10^*^	0.17^**^	(0.96)		
7. Knowledge sharing	4.65	1.23	−0.01	0.16^**^	0.04	−0.14^**^	0.20^**^	0.28^**^	(0.97)	
8. Knowledge hiding	3.24	1.50	0.05	0.07	0.03	−0.07	0.29^**^	0.29^**^	−0.02	(0.93)

*N* = 361; Internal consistency reliabilities are in parentheses; ^*^p < 0.05; ^**^p < 0.01.

#### Performance pressure

We measured performance pressure with a four-item scale developed by Mitchell et al. (2018). A sample item of this scale included: “The pressures for performance in my workplace are high.” The Cronbach’s alpha for this scale was 0.90.

#### Perceived supervisor support

Perceived supervisor support was measured using an eight-item from Eisenberger et al. (2002). Sample items included, “My supervisor strongly considers my goals and values” and “Help is available from my supervisor when I have a problem.” The Cronbach’s alpha for this scale was 0.96.

#### Knowledge sharing

Knowledge sharing was rated with an 8-item scale developed by Lu et al. (2006). Sample items included “I share with others useful work experience and know-how” and “In the workplace I take out my knowledge to share with more people” (Cronbach’s *α* = 0.97).

#### Knowledge hiding

Knowledge hiding was rated with a 3-item scale developed by Peng (2012). Sample items included “I withhold helpful information or knowledge from others” and “I do not want to transform valuable skills and expertise into organizational knowledge” (Cronbach’s α = 0.93).

## Study 1: results

### Manipulation checks

The mean values of the manipulation check for high performance pressure condition (*Mean* = 4.88, *SD* = 0.89) and the low performance pressure condition (*Mean* = 3.93, *SD* = 1.43) showed a significant difference [*F*(1, 357) = 61.84, *p* < 0.001]. In addition, the results demonstrated participants’ mean ratings of supervisor support to be higher for participants in the high supervisor support condition (*Mean* = 4.48, *SD* = 1.07) than in the low supervisor support condition [*Mean* = 3.44, *SD* = 1.43, *F* (1, 357) = 63.56, *p* < 0.001]. Our manipulations were thus effective.

### Hypotheses testing

**Figure 1:**
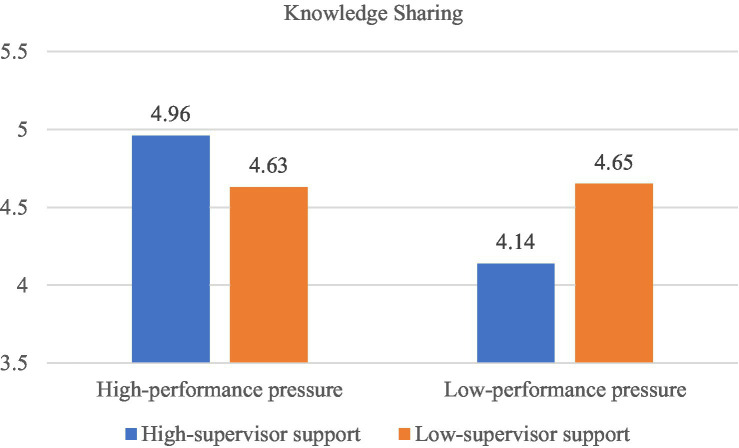
The interactive effect of performance pressure and supervisor support on knowledge sharing in Study 1.

We conducted the ANOVA test to test Hypothesis 1 and Hypothesis 2. Results from the ANOVA test showed knowledge sharing to be significantly influenced by the interaction of employee performance pressure and their perceptions of supervisor support (*F* (1, 357) = 10.78, *p* < 0.01). As illustrated in Figure 1, among participants in the high supervisor support perception condition (illustrated by the orange bars), the mean level of knowledge sharing is higher for those in the high-level performance pressure condition (*Mean* = 4.96, 95% CI = [4.74, 5.19], *n* = 113) than for those in the low-level performance pressure condition (*Mean* = 4.14, 95% CI = [3.85, 4.43], *n* = 67); and this mean difference is significant (Δ*M* = 0.82, *p* < 0.001). Additionally, among participants in the low supervisor support perception condition (illustrated by the blue bars), the mean levels of knowledge sharing for participants in the high-level performance pressure condition (*Mean* = 4.63, 95% CI = [4.40, 4.87], *n* = 101) versus low performance pressure condition (*Mean* = 4.65, 95% CI = [4.39, 4.92], *n* = 80) do not significantly differ (Δ*M* = −0.02, *p* = 0.91). Thus, consistent with Hypothesis 1, employee performance pressure interact with perceived supervisor support can impact knowledge sharing, such that performance pressure can promote knowledge sharing when supervisors show higher support.

**Figure 2:**
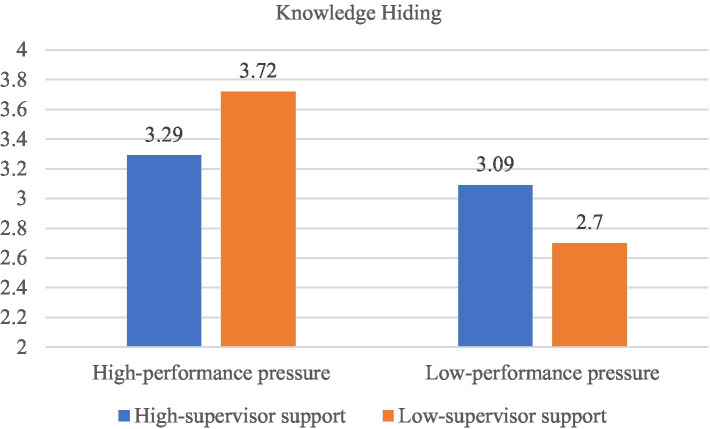
The interactive effect of performance pressure and supervisor support on knowledge hiding in Study 1.

Furthermore, results from the ANOVA test showed knowledge hiding to be significantly influenced by the interaction of employee performance pressure and their perceptions of supervisor support [*F*(1, 357) = 6.78, *p* = 0.10]. As illustrated in Figure 2, among participants in the low supervisor support perception condition (illustrated by the orange bars), the mean level of knowledge hiding is higher for those in the high-level performance pressure condition (*Mean* = 3.72, 95% CI = [2.74, 3.44], *n* = 101) than for those in the low-level performance pressure condition (*Mean* = 2.70, 95% CI = [3.43, 4.00], *n* = 80); and this mean difference is significant (Δ*M* = 1.01, *p* < 0.001). Additionally, among participants in the high supervisor support perception condition (illustrated by the blue bars), the mean levels of knowledge hiding for participants in the high-level performance pressure condition (*Mean* = 3.29, 95% CI = [3.02, 3.56], *n* = 113) versus low performance pressure condition (*Mean* = 3.09, 95% CI = [3.61, 4.23], *n* = 67) do not significantly differ (Δ*M* = 0.20, *p* = 0.38). Thus, consistent with Hypothesis 2, employee performance pressure interact with perceived supervisor support can impact knowledge sharing, such that performance pressure can promote knowledge hiding when supervisors show lower support.

## Study 2: a time-lagged field survey

### Sample and procedures

Participants in this study were full-time employees who came from a manufacturing company located in eastern China. This company specialized in manufacturing semiconductor components and utilized a piece-rate compensation system to evaluate employee performance. This payment structure imposes significant performance pressure on employees, as their working hours and remuneration are contingent upon the volume of production they achieve.

We conducted a two-wave survey with each interval lasting 4 weeks to weaken the potential impact of common method variance (Podsakoff et al., 2003). With the assistance of the HR managers of the two companies, we obtained a roster of participants and assigned each of them a unique number on the questionnaire to match responses at different time points. Meanwhile, the HR managers informed each participant that the survey was intended solely for academic research. They helped us emphasize that participation was confidential and voluntary and assured participants that the results would not be shared with their supervisors or the company. Participants who completed all surveys received a gift valued at 20 RMB as a token of appreciation.

At time point 1, 279 participants were invited to provide demographic information and their performance pressure and perceived supervisor support. A total of 246 participants responded, achieving a response rate of 88.17%. At time point 2, we invited these 246 participants to report on knowledge sharing and hiding. A total of 216 participants responded, achieving a response rate of 88.62%. After excluding 2 unmatched samples between time points 1 and 2, we obtained a final dataset of 216 employee responses, corresponding to a response rate of 77.42%. Among the participants, 36.57% of them were female; averaging 36.75 years-old (*SD* = 5.66); 37.50% had college degrees or above. They had average organizational tenures of 3.55 years (*SD* = 3.29).

### Measurements

The measures employed in Study 2 were the same as those used in Study 1. The descriptive statistics, their correlation, and Cronbach’s alpha of these variables were reported in Table 2.

**Table 2:** Descriptive statistics and correlations among study variables in Study 2.

Variables	Mean	*SD*	1	2	3	4	5	6	7	8
1. Subordinate gender	0.37	0.48								
2. Subordinate age	36.75	5.66	−0.18^**^							
3. Subordinate tenure	3.55	3.29	−0.05	0.21^**^						
4. Subordinate education	1.42	0.57	0.02	−0.27^**^	−0.06					
5. Performance pressure	4.41	1.16	0.01	0.08	0.20^**^	0.07	(0.88)			
6. Supervisor support	3.79	1.05	0.01	−0.06	0.04	0.12	0.01	(0.74)		
7. Knowledge sharing	4.00	1.03	0.01	0.04	0.01	0.04	0.01	0.01	(0.76)	
8. Knowledge hiding	4.12	1.30	−0.07	−0.02	−0.05	0.06	0.08	−0.15^*^	−0.13	(0.80)

*N* = 216; Internal consistency reliabilities are in parentheses; ^*^p < 0.05; ^**^p < 0.01.

#### Control variables

To rule out other potential influences on the results of other exogenous factors, we included employee age, gender, education level, organizational tenure, and work hours per week as controls as previous studies suggested (Gerpott et al., 2020; Nerstad et al., 2018). Age and tenure were self-reported in years. Work hours per week were self-reported in hours. Gender was coded as “0 = female” and “1 = male.” Education was coded as “1 = below college degree,” “2 = college degree,” “3 = bachelor’s degree,” and “4 = master’s degree.”

## Study 2: results

### Confirmatory factor analyses

We conducted serval CFAs to evaluate the appropriateness of our measurement model. The results showed that four-factor model fit the data well [*χ^2^*_(224)_ = 271.85, CFI = 0.96, TLI = 0.96, RMSEA = 0.03, SRMR = 0.05], and better than a three-factor model in which performance pressure and supervisor support were combined into one factor [*χ^2^*_(227)_ = 519.85, CFI = 0.75, TLI = 0.73, RMSEA = 0.08, SRMR = 0.10, ∆*χ^2^*_(∆*df* = 3)_ = 248.00, *p* < 0.00]; and a single-factor model in which all the variables were combined into one factor [*χ^2^*_(230)_ = 983.41, CFI = 0.37, TLI = 0.30, RMSEA = 0.12, SRMR = 0.14, ∆*χ^2^*_(∆*df* = 6)_ = 711.56, *p* < 0.00]. Thus, the four variables in the conceptual model were distinctive constructs.

### Hypotheses testing

**Figure 3:**
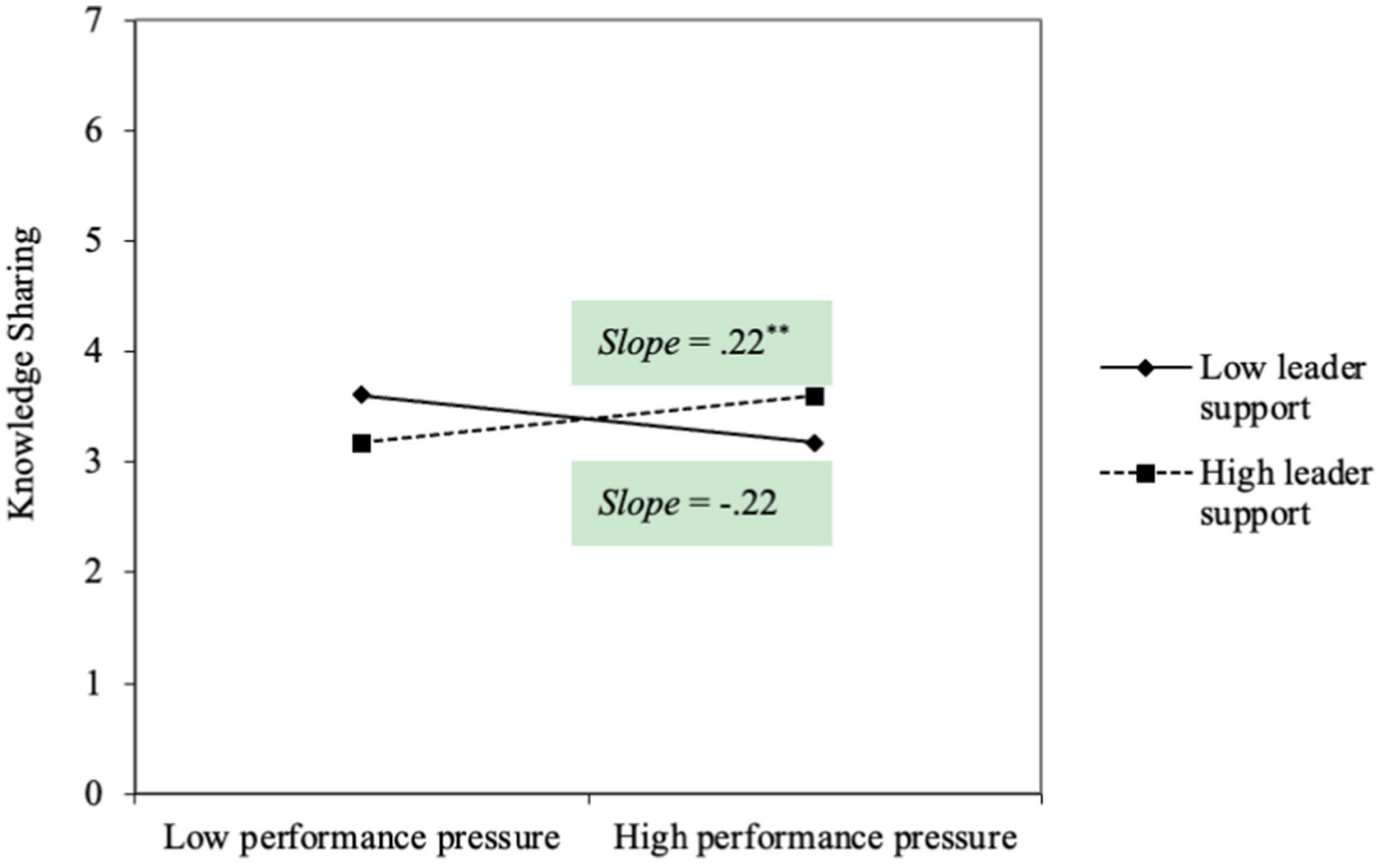
The interactive effect of performance pressure and supervisor support on knowledge sharing in Study 2.

**Figure 4:**
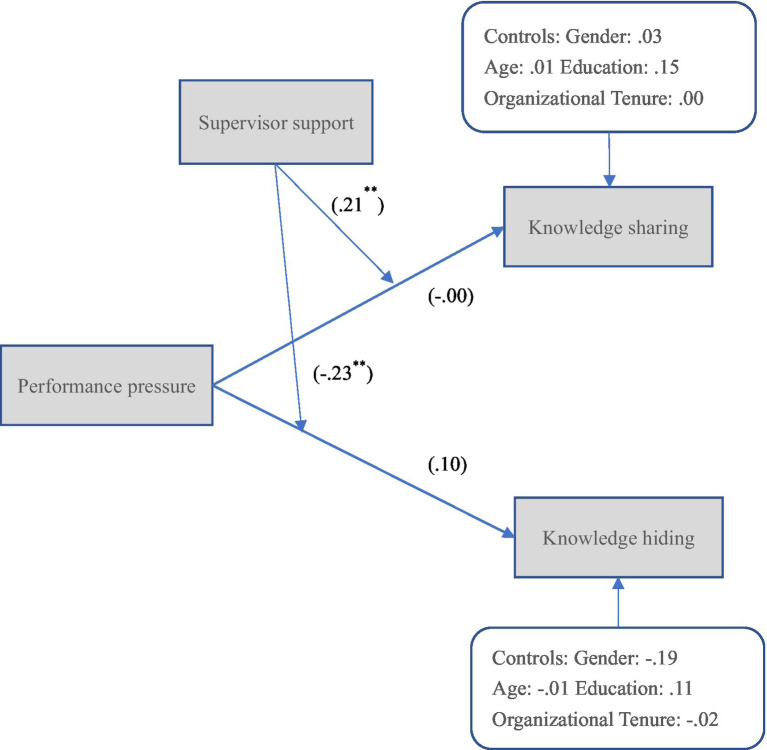
The path analytic results in Study 2.

Hypothesis 1 proposed that performance pressure predicted knowledge sharing under the high level of perceived supervisor support. As shown in Figure 3, the interactive effect of performance pressure and perceived supervisor support was positively and significantly related to knowledge sharing (*b* = 0.21, *s.e.* = 0.08, *p* < 0.01), Hypothesis 1 was supported. Hypothesis 2 proposed that performance pressure predicted knowledge hiding under a low level of perceived supervisor support. As shown in Figure 4, the interactive effect of performance pressure and perceived supervisor support was negatively and significantly related to knowledge hiding (*b* = −0.23, *s.e.* = 0.08, *p* < 0.01), Hypothesis 2 was supported.

**Figure 5:**
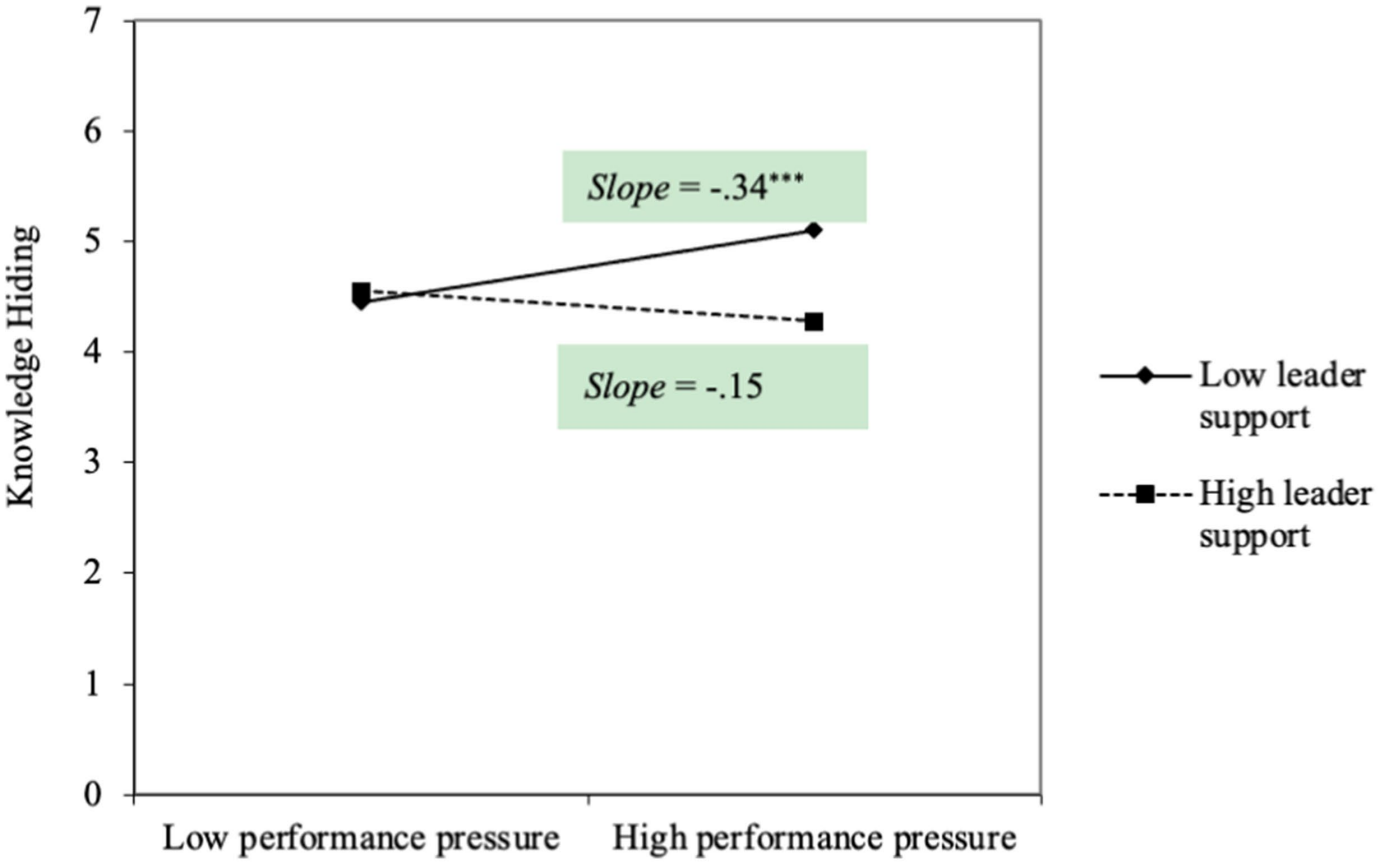
The interactive effect of performance pressure and supervisor support on knowledge hiding in Study 2.

To further describe the interactive effects, we estimated the simple slopes and plotted the significant interactions at 1 *SD* above and below the mean for perceived supervisor support. As shown in Figure 5, performance pressure had a positive impact on knowledge sharing when perceived high level of supervisor support (*b* = 0.22, *s.e.* = 0.09, *p* < 0.05), while it becomes nonsignificant under low level of perceived supervisor support (*b* = −0.22, *s.e.* = 0.13, *p* = 0.08). As shown in Figure 3, performance pressure had a positive impact on knowledge hiding when perceived low level of supervisor support (*b* = 0.34, *s.e.* = 0.10, *p* < 0.00), while it becomes nonsignificant under high level of perceived supervisor support (*b* = −0.15, *s.e.* = 0.14, *p* = 0.28). Thus, our hypotheses were both further supported.

## Discussion

This study validates a dual-path model of performance pressure on employees’ knowledge-sharing and knowledge-hiding behaviors through a scenario experiment (Study 1) and a multi-wave survey (Study 2). The findings indicate that performance pressure facilitates knowledge sharing when supervisors provide higher support, whereas it can lead to increased knowledge hiding behavior when supervisor support is low. These results reveal the complex impact of performance pressure on knowledge management behaviors under different circumstances and offer new insights into understanding the relationship between performance pressure, supervisor support, and knowledge management behaviors.

### Theoretical implications

Firstly, this research combines employees’ internal pressure perception with external supervisor support to reveal the conditions under which employees engage in different knowledge management behaviors. Our findings suggest that for employees experiencing performance pressure, adequate supervisor support equals resource guarantees that provide conditions for knowledge sharing, while in the absence of support, performance pressure may prompt employees to adopt protective strategies and reduce information flow. Previous studies have focused on factors such as workplace stressors as drivers that promote or inhibit knowledge sharing and knowledge hiding (He et al., 2024; Li et al., 2023; Mahapatra and Ford, 2024; Shujahat et al., 2024; Skerlavaj et al., 2018; Sofyan et al., 2023; Zhang et al., 2022). Although some have clarified the possible boundary conditions of these effects, our understanding of the conditions for the generation of knowledge management behavior is still somewhat limited. By combining the dual perspectives of resource depletion and resource enrichment (Halbesleben et al., 2014; Hobfoll, 1989; Hobfoll et al., 2018; Lin et al., 2020), this study reveals that the impacts of performance pressure on knowledge management behaviors depend on the specific context factors in which employees are located, rather than being driven solely by performance pressure itself. In doing so, we further clarify how employees choose knowledge management strategies under performance pressure, that is performance pressure does not directly lead to a decrease or an increase in knowledge sharing and hiding. Instead, its effects are determined by external situational factors (supervisory support) and performance pressure. Overall, our study reveals that the generation of knowledge sharing, and knowledge hiding does not occur necessarily, but is the result of the combined effects of multiple variables such as workplace stressors and supportive resources in the context, thus providing new insights into the relationship between performance pressure and knowledge management behavior.

Secondly, this study expands our understanding of drivers for knowledge sharing and knowledge hiding by examining the interaction between high supervisor support and performance pressure. While existing knowledge management literature has explored various stressors, such as workplace demands (He et al., 2024; Montani et al., 2024; Skerlavaj et al., 2018; Sofyan et al., 2023; Zhang et al., 2022), and various managerial drivers (Cheng and Chen, 2024; Kim and Lee, 2006; Koay and Lim, 2022; Reinholt et al., 2011; Serenko and Bontis, 2016; Srivastava et al., 2006; Watkins et al., 2023) such as top management support, cultural climate, and reward systems. This study departs from traditional knowledge management frameworks and finds that the global (generalized) expectation of rewards or punishments associated with performance pressure serves as a potential factor in resource enrichment or depletion, which can be activated or mitigated by supervisor support, ultimately influencing employees’ knowledge-sharing or hiding behaviors. In doing so, our findings contribute to a more nuanced understanding of the antecedents of knowledge management behaviors by integrating both stressors and managerial element (Connelly et al., 2012; Liao et al., 2024).

Finally, this study provides additional evidence for the paradoxical nature of performance pressure. Specifically, by incorporating both resources enrichment and resources depletion paths into the analysis, our study broadens the resource-based perspective on how employee performance pressure influences knowledge management outcomes. Previous research has predominantly examined the effects of employee performance pressure either through the lens of resources enrichment path (Guo et al., 2024; Kundi et al., 2022; Mitchell et al., 2019) or resources depletion path (Chen and Chen, 2023; Wang et al., 2025; Zhu et al., 2023) in isolation. However, considering that pressure can serve as a motivator for employees to acquire resources while also deplete their existing resources, an exclusive emphasis on resources enrichment or depletion path provides a limited, and potentially misleading perspective on the function of performance pressure (Spoelma, 2022). Drawing on COR theory, we introduce the concepts of knowledge sharing (a resource enrichment path) and knowledge hiding (a resource depletion path) as outcomes of interaction between employee performance pressure and perceived supervisor support. This attempts not only provide a comprehensive understanding of how employee performance pressure operates by highlighting the multifaceted resources path involved, but also emphasize the paradoxical role of performance pressure as both a motivator and an obstructor for employees’ knowledge management behavior.

### Practical implications

Based on the findings of this study, we propose targeted practical recommendations to guide organizational managers in optimizing employee knowledge management behaviors under high-performance pressure conditions.

Firstly, optimizing supervisor support strategies to address the dual-edged effect of performance pressure. This study highlights that performance pressure can either promote knowledge sharing or lead to knowledge hiding, depending on the level of supervisor support. To maximize the positive effects and minimize the negative impacts of performance pressure, organizations should focus on optimizing supervisor support strategies. Providing high levels of supervisor support can stimulate employees’ proactive social reciprocity motives, making them more willing to respond to performance pressure in ways that benefit the organization. For instance, specialized training can be provided to supervisors at all levels to help them understand how to offer effective emotional and resource support under high-performance pressure. Additionally, organizational policies can be developed to enhance employees’ perceptions of supervisor support, such as regular communication meetings to encourage employees to express their needs and concerns, thereby increasing targeted support and assistance from supervisors (Liden et al., 2008; Malik et al., 2019).

Secondly, this study offers insights for managers to enhance knowledge sharing within the organization. Treating performance pressure as a potential factor in social exchange can activate employees’ social exchange motives, thereby promoting knowledge sharing. Managers can leverage this mechanism to design incentive systems and management strategies that boost employees’ willingness to share knowledge. For example, establishing clear and fair reward mechanisms ensures that employees’ efforts are adequately recognized. Furthermore, fostering a supportive work environment, such as creating a culture that emphasizes cooperation over competition, can make employees feel that their knowledge contributions are valued.

Lastly, this study provides new perspectives for managers to effectively identify and eliminate factors leading to knowledge hiding behaviors. The study suggests that high-performance pressure can lead to knowledge hiding when supervisor support is insufficient. Therefore, organizations need to identify the triggers of knowledge hiding behaviors and take measures to alleviate them. For example, regularly assessing job characteristics and leadership behaviors can reduce employees’ negative reciprocity beliefs by providing adequate supervisor support, thereby mitigating the process of knowledge hiding being triggered. Additionally, cultivating a positive organizational support atmosphere, where employees feel supported and respected by the organization, is an effective way to reduce knowledge hiding behaviors.

### Limitations and future research directions

While our study has provided valuable insights, there are certain limitations that future research needs to address. Firstly, we measured variables using individual self-reports, which may lead to common method bias. Therefore, future research could explore alternative approaches, such as measuring knowledge management behaviors (i.e., sharing and hiding) through supervisor or coworker ratings. Furthermore, although we used a multi-wave survey approach and performed a scenario experiment to minimize common method bias and promote internal validity, our data remained cross-sectional. Thus, future research could consider longitudinal studies to capture more dynamic processes.

Secondly, our findings indicated that the direct effects of performance pressure on knowledge sharing and hiding were nonsignificant; hence, we explored the interactive effect of performance pressure and supervisor support from a COR perspective. Future research should consider introducing indirect roles to explore the influencing mechanisms under another theoretical framework, such as social exchange, between performance pressure and knowledge management behaviors.

Finally, while we mainly examined perceived supervisor support as a moderating variable for the relationship between performance pressure and knowledge sharing and hiding behaviors, the conceptual model has been supported both theoretically and empirically. However, it is essential to acknowledge the presence of other potential boundary conditions within different theoretical frameworks. Future research endeavors can delve into exploring additional factors that could act as boundary conditions, such as organizational culture, team interactions, and personal values, all of which have the potential to influence the relationship between employee performance pressure and knowledge management behaviors in significant ways.

## Data Availability

The raw data supporting the conclusions of this article will be made available by the authors, without undue reservation.
