# Supplementary material for: To share or hide under performance pressure: the role of supervisor support in shaping subordinate knowledge management behaviors
Source: Front Psychol. 2025 May 27;16:1586812. doi: 10.3389/fpsyg.2025.1586812 (PMC12149133; doi:10.3389/fpsyg.2025.1586812)
Supplement: Supplementary file 1 [file Data_Sheet_1.pdf]

Appendix

High-performance pressure

Zhang Feng is a software development employee at a renowned high-tech company, involved in a novel project with significant market potential. This project is highly prioritized within the company, regarded as a critical milestone for its development in the coming years. In this context, Zhang Feng experiences a profound sense of responsibility and pressure. He recognizes that the project's success would not only be highly beneficial to the company but could also present opportunities for his personal career advancement. As the project progresses, Zhang Feng increasingly feels the strain of work-related pressure. He begins to worry that even minor errors could lead to the project's failure. Overtime has become a routine part of his work, and he frequently spends late nights addressing technical challenges. Internally, he is consumed by anxiety, fearing that if the project fails to meet expectations, his performance within the company may be negatively evaluated.

Low-performance pressure

Zhang Feng is a software development employee at a renowned high-tech company, involved in a novel project with significant market potential. This project is highly prioritized within the company, regarded as a critical milestone for its development in the coming years. Despite its significance, the company adopts a more relaxed and supportive management approach. It encourages Zhang Feng to focus on technical growth and innovation, without placing undue emphasis on the project's success or

failure. The company prioritizes employees' ability to enhance their skills and competencies through the project, assuring that even if the project fails, no excessive penalties will be imposed. Consequently, Zhang Feng experiences enthusiasm for technology and a relaxed work environment, rather than an urgent pressure to achieve high performance.

High-Leader support

During a critical phase of the project, Zhang Feng encountered technical and coordination challenges. In this context, his leader demonstrated exceptional support. The leader not only frequently inquired about Zhang Feng's progress and potential difficulties but also provided substantial assistance and resources. When Zhang Feng felt anxious about a minor error, the leader reassured him, saying, "It's alright; we all make mistakes. What matters is that we learn from them." The leader consistently encouraged Zhang Feng to share his insights and publicly acknowledged his contributions during departmental meetings, ensuring that Zhang Feng felt his work was highly valued. Furthermore, the leader is considering recommending Zhang Feng for a leadership role in future projects.

Low-Leader support

Despite the project entering a critical phase, Zhang Feng feels increasingly sidelined. His leader appears to show limited concern for the project's progress. When Zhang Feng attempts to discuss the challenges he faces, his leader often responds hurriedly,

offering little concrete assistance. On one occasion, Zhang Feng was harshly criticized by his leader for a minor mistake, leaving him deeply disheartened. The leader rarely solicits Zhang Feng’s opinions or acknowledges his contributions during meetings, resulting in Zhang Feng feeling marginalized within the team. Moreover, when discussing potential leaders for future projects, the leader seems to have never considered Zhang Feng.
