# Supplementary material for: To share or hide under performance pressure: the role of supervisor support in shaping subordinate knowledge management behaviors
Source: Front Psychol. 2025 May 27;16:1586812. doi: 10.3389/fpsyg.2025.1586812 (PMC12149133; doi:10.3389/fpsyg.2025.1586812)
Supplement: Supplementary file 2 [file Supplementary_file_1.pdf]

English version

Ethical Statement (Approval)

Research Title: Study on Workplace Pressure, Employees Behavior, and Performance
Date: Jan 2024 - Sep 2024

1. Research Overview

This study aims to investigate the impact of workplace performance pressure on employee behaviors. The research methods include scenario-based experiments (simulating workplace scenarios), field experiments (conducted in real work environments), and questionnaire surveys. The study involves the following two participant groups: (1) Part-time undergraduate students; (2) manufacturing enterprise employees. The study is scheduled to take place from Jan 2024 to Sep 2024 at a Manufacturing enterprise in Eastern of China and a public university in Southwest China.

2. Ethical Principles

This study adheres to the ethical principles outlined in the Declaration of Helsinki and the Regulations on Ethical Review of Biomedical Research Involving Human Subjects (China, 2016). It is committed to protecting the rights, safety, and well-being of participants, ensuring compliance with the principles of respect for persons, beneficence, and justice.

3. Informed Consent

All participants will receive an informed consent form prior to participation, detailing the study's purpose, procedures, potential risks, benefits, and the right to withdraw voluntarily. The informed consent form will be provided in Chinese to ensure full comprehension. Part-time undergraduate students will provide consent through written or online confirmation; manufacturing enterprise employees will provide written consent before participation. Participants may withdraw from the study at any time without any adverse consequences.

4. Risks and Benefits

(1) Potential Risks: This study is low risk. Scenario-based and field experiments may involve minimal psychological stress (e.g., simulating performance evaluation scenarios) but will not cause physical or long-term psychological harm. The questionnaire is anonymous and involves only general work-related questions, excluding sensitive personal information.
(2) Risk Management: Experimental scenarios will be designed as low-stress environments to avoid discomfort. Questionnaires will be fully anonymous, with no collection of identifiable information (e.g., names, ID numbers). Contact details of the research team will be provided for participants to seek support or provide feedback.
(3) Potential Benefits: The study's findings will provide data to optimize workplace performance policies, potentially benefiting the participants' organizations or similar

institutions.

5. Privacy and Data Protection
All data will be collected anonymously, with no identifiable personal information included. Experimental and questionnaire data will be stored on an encrypted cloud server, accessible only to authorized research team members. Research outputs (e.g., papers or reports) will present only aggregated data, ensuring no individual information is disclosed.

6. Additional Considerations
For manufacturing enterprise employees, who may be in hierarchical workplace relationships, the research team will explicitly state that participation is entirely voluntary, and data will not be shared with their employers. For part-time undergraduate students, the study will be conducted in a manner that does not interfere with their regular studies. If participants experience discomfort during experiments or surveys, they may contact the research team for support or to withdraw from the study.

7. Declaration
I pledge that this study will strictly adhere to the ethical principles outlined above and will be subject to oversight by the Department of Education Evaluation and Supervision, Chengdu University of Technology. This study has received ethical approval from the Department of Education Evaluation and Supervision, Chengdu University of Technology (Contact number: +86 02884077647). Any ethical issues will be promptly reported and addressed by the research team.

Researcher's Signature: Jiou Xu

Institutional Approval: 成都理工大学 教育评价与督导处

Chinese version

伦理声明

研究题目：员工工作场所压力、行为和绩效研究

日期：2024 年 1 月-2024 年 9 月

1. 研究概述

本研究旨在探讨工作场所的绩效压力对员工行为的影响。研究方法包括情景实验（模拟工作场景）和田野实验（基于真实工作环境），并辅以问卷调查。研究对象包括以下两类群体：（1）非全日制本科生（2）制造业企业员工。

研究预计于 2024 年 1 月至 2024 年 9 月进行，地点为中国东部地区某制造业企业、西南地区公立高校。

2. 伦理原则

本研究遵循《赫尔辛基宣言》和《涉及人的生物医学研究伦理审查办法》（中国，2016 年）的伦理原则，致力于保护受试者的权益、安全和福祉，确保研究过程符合尊重个人、善行和公正的原则。

3. 知情同意

所有受试者将在参与研究前收到知情同意书，内容包括研究目的、实验程序、潜在风险、益处及自愿退出权利。知情同意书将以中文提供，确保受试者能够充分理解。非全日制本科生将通过书面或在线确

认形式签署知情同意书；制造业企业员工将在实验前通过书面形式签署。受试者可随时无条件退出研究，且不会因此受到任何不利影响。

4. 风险与益处

潜在风险：本研究为低风险研究。情景实验和田野实验可能涉及轻微的心理压力（如模拟绩效评估场景），但不会对受试者造成身体或长期心理损害。问卷调查为匿名形式，仅涉及一般工作相关问题，不包含敏感个人信息。

风险管理：实验场景将设计为低压力环境，避免引发不适。问卷调查将完全匿名，不收集可识别个人信息（如姓名、身份证号）。研究团队将提供联系方式，供受试者在需要时咨询或反馈。

潜在益处：研究结果将为优化工作场所绩效政策提供数据支持，可能为受试者所在机构或类似组织带来管理改进。

5. 隐私与数据保护

所有数据以匿名形式收集，不包含任何可识别受试者身份的信息。实验数据和问卷数据将存储于加密的云端服务器，仅限研究团队授权成员访问。任何研究成果（如论文或报告）将仅呈现汇总数据，不会披露任何个体信息。

6. 其他考虑

对于制造业企业员工，为避免潜在压力，研究团队将明确告知其参与完全自愿，且实验数据不会与雇主共享。对于非全日制本科生，

本研究不干扰正常学习。若受试者在实验或问卷中感到不适，可随时联系研究团队，获取支持或退出研究。

7. 声明

本人承诺本研究将严格遵循上述伦理原则，并接受成都理工大学教育评价与督导处的监督。本研究已获得成都理工大学教育评价与督导处的伦理许可（联系电话：+86 02884077647），如有任何伦理问题，研究团队将及时报告并采取纠正措施。

研究者签名：[illegible]

学院审批：

成都理工大学 教育评价与督导处
